# A long-term survivor of hilar cholangiocarcinoma with resection of recurrent peritoneal dissemination after R0 surgery: a case report

**DOI:** 10.1186/s40792-017-0386-z

**Published:** 2017-10-16

**Authors:** Tatsunori Miyata, Hirohisa Okabe, Akira Chikamoto, Takanobu Yamao, Naoki Umezaki, Masayo Tsukamoto, Yuki Kitano, Kota Arima, Shigeki Nakagawa, Katsunori Imai, Daisuke Hashimoto, Yo-ichi Yamashita, Hideo Baba

**Affiliations:** Department of Gastroenterological Surgery, Graduate School of Life Sciences, Kumamoto University, 1-1-1 Honjo, Chuo-ku, Kumamoto, 860-0811 Japan

**Keywords:** Chemotherapy, Hilar cholangiocarcinoma, Long survivor, Recurrence, Peritoneal dissemination

## Abstract

**Background:**

Although hilar cholangiocarcinoma (HCCA) has a very poor prognosis, there are cases in which long-term survival is rarely obtained by multidisciplinary treatment.

**Case presentation:**

A 61-year-old man diagnosed with HCCA was referred to our hospital. We performed an extended left hemi-hepatectomy and caudate lobectomy with extrahepatic bile duct resection. The tumor stage was T2aN0M0, stage II, based on the TNM classification, seventh edition. R0 resection was successfully performed. Adjuvant chemotherapy was not administered. After 38 months, computed tomography revealed peritoneal dissemination. The patient received chemotherapy with tegafur-gimeracil-oteracil-potassium (S-1) and gemcitabine. The peritoneal dissemination was successfully controlled for more than 50 months. During the treatment, levels of CEA and CA19-9 kept rising slowly, which was followed by bowel obstruction due to peritoneal dissemination of HCCA. The patient underwent resection of transverse colon with tumor nodules, and the tumor was pathologically diagnosed as metastasis of HCCA. Tumor markers decreased to normal levels, and the patient has been free from tumor relapse for 6 months.

**Conclusions:**

We here report a rare case of HCCA patient with recurrent peritoneal dissemination 3 years after R0 surgery which was sensitive to chemotherapy. The patient successfully received resection of peritoneal dissemination 50 months after the induction of chemotherapy and survived for 10 years.

## Background

Hilar cholangiocarcinoma (HCCA) is a type of bile duct cancer that occurs in the extrahepatic biliary tree proximal to the origin of the cystic duct [1]. It is the most common malignancy arising from the biliary tract, accounting for two thirds of cholangiocarcinoma [2]. The prognosis is generally poor because early diagnosis is difficult and most patients present with advanced disease. Unfortunately, only 25% of the patients present resectable tumors at the time of diagnosis. A complete surgical resection remains the only option for a cure; and the subsequent 5-year survival rate ranges from 11 to 42% [3]. Moreover, in the case of peritoneal dissemination of HCCA, the prognosis is even worse [4]. To improve the prognosis, resection of local disease is sometimes effective together with multidisciplinary treatments such as chemotherapy, radiation therapy, or chemoradiotherapy before or after surgery [5, 6]. However, there are few reports of long survivor of HCCA patients with recurrent disease of peritoneal dissemination after curative surgery [7].

We here report a rare case that chemotherapy could control the recurrence of peritoneal dissemination of HCCA over 50 months which was resected successfully and survived more than 10 years after initial resection with normal tumor marker level.

## Case presentation

**Fig. 1:**
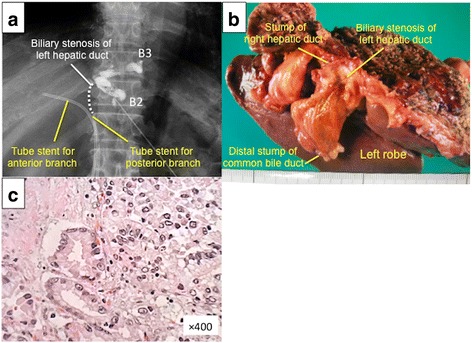
Initial resection of primary tumor. **a** Left hepatic duct was obstructed (dotted line), and a biliary stent was placed both in the anterior branch and the posterior branch. **b** Surgical resected specimen is shown. Stump of the right hepatic duct is free from tumor spreading. **c** Hematoxylin and eosin staining indicates moderately differentiated tubular adenocarcinoma

A 61-year-old man, who was diagnosed with HCCA after an endoscopic retrograde biliary drainage for obstructive jaundice, was referred to our hospital. Laboratory tests showed that the patient’s total bilirubin, aspartate transaminase, alanine aminotransferase levels were 1.2 mg/dl, 56 U/L, and 161 U/L, respectively. Levels of carcinoembryonic antigen (CEA) and carbohydrate antigen 19-9 (CA 19-9) were 3.3 ng/mL and 40.4 U/mL, respectively. Percutaneous transhepatic bile duct drainage for the left branch of intrahepatic bile duct and cholangiography showed that biliary tract was obstructed at the hilum (Fig. 1a). Imaging study indicates that the tumor was classified as type IIIB HCCA according to the Bismuth-Corlette classification system (data not shown) [8]. The patient received an extended left hemi-hepatectomy and caudate lobectomy with lymphadenectomy and extrahepatic bile duct resection (Fig. 1b). Proximal margin of the right hepatic duct and distal margin of the common bile duct were negative for malignancy. A reconstruction was performed with Roux-en-Y retro-colic hepaticojejunostomy. The pathological findings revealed moderately differentiated tubular adenocarcinoma (Fig. 1c). There were no lymph node metastasis and vascular invasion. The pathological tumor staging was stage II, T2aN0M0, according to the UICC/AJCC seventh edition staging system [9]. After the operation, he recovered and was discharged without any complications.

**Fig. 2:**
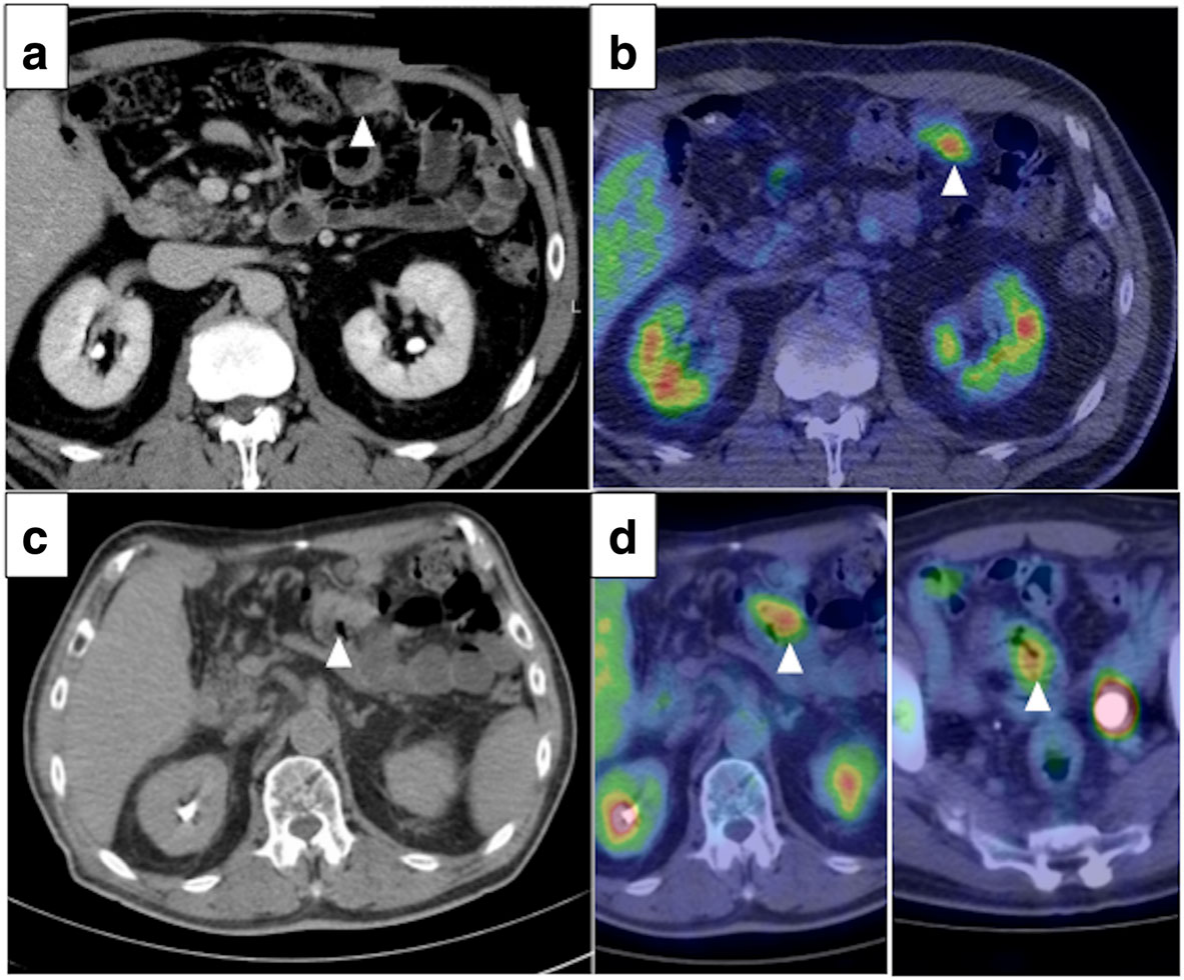
Recurrence of peritoneal dissemination. Peritoneal dissemination was detected by CT (**a**) and positron emission tomography-CT (PET-CT) (**b**). Bowel obstruction by the tumor was detected by CT (**c**) and PET-CT (**d**). Arrowheads show the peritoneal disseminations

**Fig. 3:**
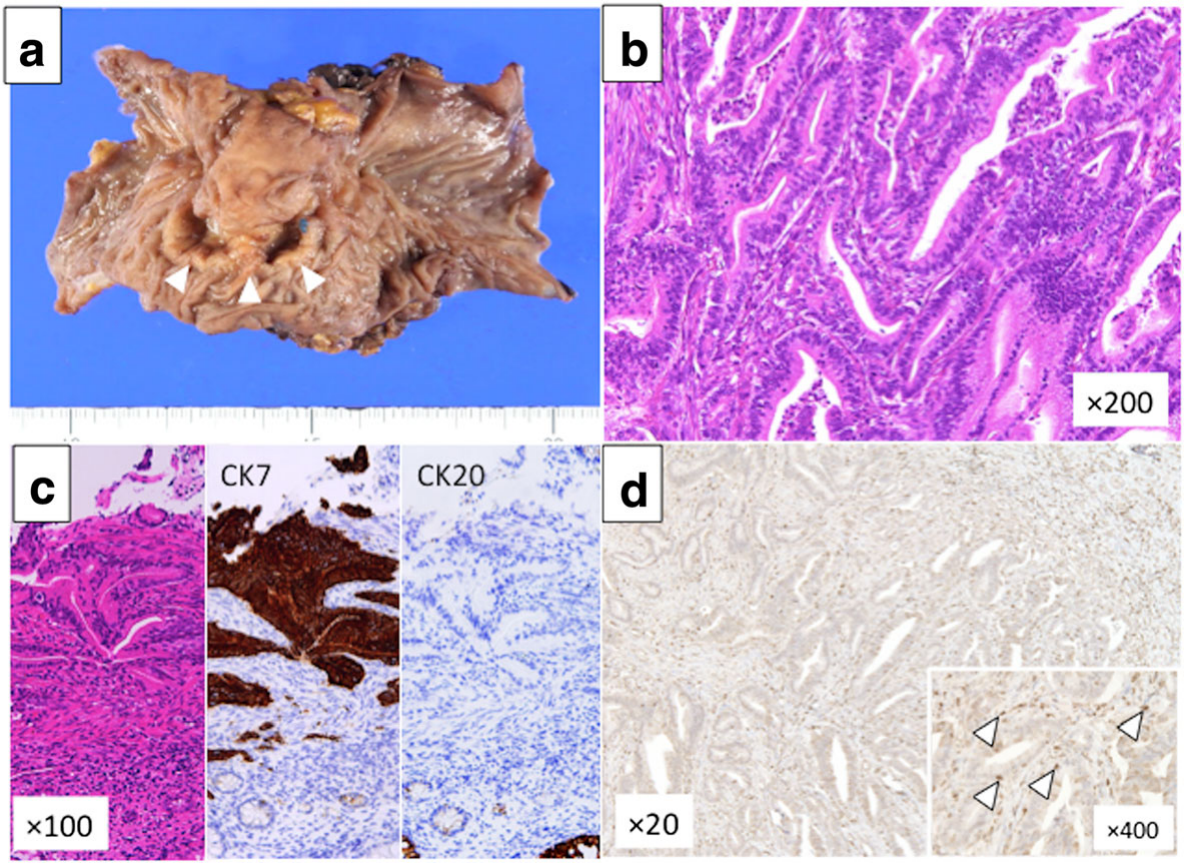
Local resection of the transverse colon with peritoneal dissemination of the hilar cholangiocarcinoma. **a** Figure shows surgical resected specimen which performed with local resection of the transverse colon. Arrowheads show the peritoneal dissemination. **b** HE staining of the recurrent tumor is shown, and the tumor was pathologically diagnosed as metastasis of HCCA of which tumor grade was moderately differentiated. **c** CK7 is positive, and CK20 is negative in immunohistochemistry. **d** Abundant CD8^+^ T cells (arrowheads) were seen in tumor stroma

**Fig. 4:**
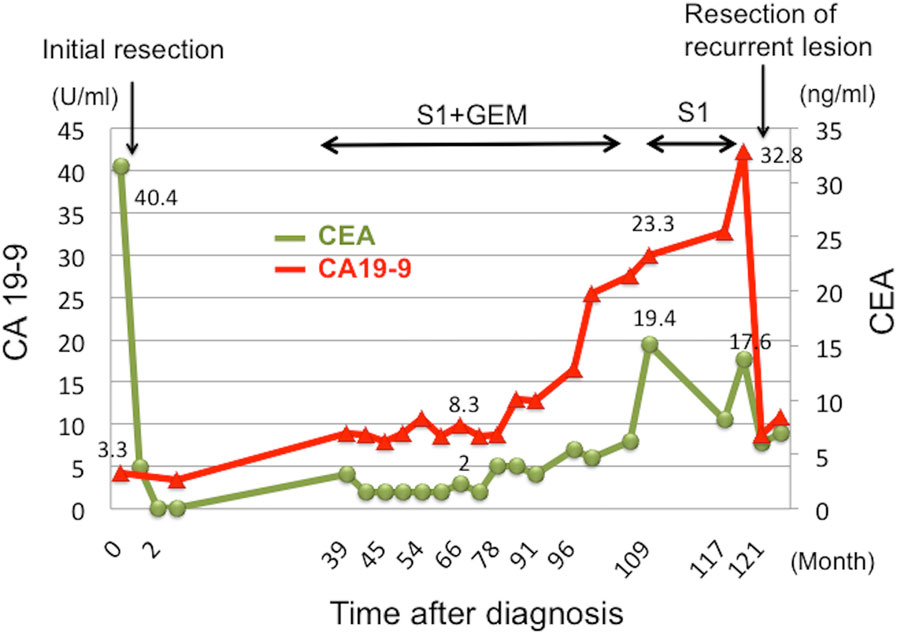
The clinical course of tumor markers. CEA and CA19-9 levels slightly increased at the recurrence of hilar cholangiocarcinoma and kept low levels for more than 6 years. After the resection of the recurrent tumor, CEA and CA19-9 decreased to almost normal levels

Thirty-eight months after the operation, CEA and CA19-9 levels had elevated, and peritoneal dissemination was detected by enhanced CT (Fig. 2a) and positron emission tomography-CT (PET-CT) (Fig. 2b). The patient received chemotherapy with tegafur-gimeracil-oteracil-potassium (S-1) and gemcitabine (GEM). Chemotherapy controlled the recurrence of peritoneal dissemination of HCCA well over 50 months. Since he had grade 3 appetite loss, the amount of chemotherapy gradually decreased and he decided to take only S-1 70 months after the initial chemotherapy. Therefore, tumor markers gradually increased, and bowel obstruction occurred 117 months after the initial surgery (Fig. 2c, d). Since the recurrent tumor was localized and obstruction needs surgical approach, the patient received the resection of the transverse colon with peritoneal dissemination. Two localized tumor nodules were resected individually. These tumors were diagnosed pathologically as metastasis of well-differentiated HCCA since immunohistochemical staining revealed that the disseminated tumors were CK7 positive and CK20 negative (Fig. 3a–c). In addition, abundant CD8^+^ T cells were found in the tumors (Fig. 3d). After the resection of the metastases, CEA and CA19-9 dropped to almost normal levels (Fig. 4). The patient was discharged without any complications and has been free from recurrent disease for 6 months.

## Discussion

Although surgical resection is the only curative treatment for HCCA, the recurrence rate after surgery is 50–75% [10]. Therefore, the response to treatment for recurrent tumors has a great impact on prognosis. There are several reports of HCCA patients who survived long through a multidisciplinary treatment. Poor prognostic factors have been reported in HCCA, such as age, preoperative high CA19–9, positive margin status, lymph node metastasis, histological grade, and advanced tumor stage [11–13]. In our case, R0 resection was performed, and there was no lymph node metastasis. The preoperative levels of CA19-9 were within the normal range at the initial resection. As reported in other cancers, re-resection for recurrence could be effective for a limited number of patients with hilar cholangiocarcinoma. Table 1 shows the summary of those cases. Briefly, Ota et al. reported a case of bone resection 10 years after initial curative surgery [14]. Yamada et al. reported 9 patients with resection of pulmonary metastasis, and the 3-year survival rate after the resection was 40.0% whereas the group without surgery showed 8.7% [15]. Koizumi et al. reported a case of resection of peritoneal dissemination after curative resection [16]. Thus, there are patients whose prognosis is improved by re-resection, although the number of such patients is very limited. Predictive factors for patients with recurrent lesions, who could benefit from curative resection, should be determined in the future.

**Table 1:** The cases for which resection was performed for recurrence of hilar cholangiocarcinoma

Case report author	Year	Age	Sex	R status in initial resection	Relapse-free survival from initial resection	Recurrence site	Treatment after recurrence
Ota et al. [14]	2013	61	F	R0	10 years	Bone	Surgery alone
Yamada et al. [15]	2017	43–74	M:7 F:2	R0: 8 R1: 1	1.3–6.8 years	Lung	Surgery + adjuvant chemotherapy/radiotherapy^a^
Koizumi et al. [16]	2016	76	M	R0	5.8 years	Urinary bladder	Chemotherapy (GEM + CDDP) + surgery
Present case	2017	61	M	R0	3.2 years	Peritoneal dissemination	Chemotherapy (GEM + S1) + surgery

Abbreviations: *GEM* gemcitabin, *CDDP* cisplatin, *RFS* relapse-free survival

^a^Six of 9 patients underwent chemotherapy/radiotherapy

Adjuvant chemotherapy has not been standardized for cholangiocarcinoma, although GEM plus cisplatin is effective for advanced cholangiocarcinoma [7]. Sasaki et al. reported that human equilibrative nucleoside transporter 1 (hENT1) is a biomarker to predict the effectiveness of chemotherapy based on GEM in bile duct cancer. Expression of hENT1 expression is associated with a well-differentiated type of tumor [17]. The recurrent tumor in our patient was also well-differentiated. Tumor microenvironment also affects susceptibility to anticancer drugs. CD8^+^ T cells play an important role in anti-tumor immunity during tumor progression [18, 19]. Several reports showed that tumor-infiltrating T cell including CD8^+^ T cell after chemotherapy was a predictor of good response to chemotherapy [20–22]. This suggested that chemotherapy triggers immunologic anti-cancer effect which influences the success of treatment for cancers. Therefore, CD8^+^ T cell accumulation in cancers can lead to better clinical outcome [23]. We also found that a risk-signature including CD8^+^ T cell status predict prognosis and sensitivity to chemotherapy after curative resection in extrahepatic cholangiocarcinoma [24]. We detected abundant CD8^+^ T cells within the tumor by immunohistochemistry, which might have contributed to the positive response to chemotherapy and long-survival.

## Conclusions

We here report a rare case of HCCA patient with recurrent peritoneal dissemination which occurred 38 months after curative surgery and successfully resected 50 months after the induction of chemotherapy. As a result, the patient survived more than 10 years after initial resection maintaining normal tumor marker level. Persistent chemotherapy seemed effective for a long period in this case, and we need to explore predictive factors for such successful chemotherapy based on the pharmaceutical mechanism of each chemotherapeutic agent.

## Abbreviations

CA19-9: Carbohydrate antigen 19-9
CEA: Carcinoembryonic antigen
CT: Computed tomography
GEM: Gemcitabine
HCCA: Hilar cholangiocarcinoma
hENT1: Human equilibrative nucleoside transporter 1
S-1: Tegafur-gimeracil-oteracil-potassium
